# DichroPipeline: A suite of online and downloadable tools and resources for protein circular dichroism spectroscopic data analyses, interpretations, and their interoperability with other bioinformatics tools and resources

**DOI:** 10.1002/pro.4817

**Published:** 2023-12-01

**Authors:** Robert W. Janes, B. A. Wallace

**Affiliations:** ^1^ School of Biological and Behavioural Sciences Queen Mary University of London London UK; ^2^ School of Biological Sciences Birkbeck University of London London UK

**Keywords:** circular dichroism (CD) spectroscopy, data processing, GitHub repository, intrinsically‐disordered proteins (IDPs), membrane proteins, online tools and resources, Protein Circular Dichroism Data Bank (PCDDB), protein structure, secondary structure analyses, soluble proteins, spectral displays and comparison methods, validation procedures

## Abstract

Circular Dichroism (CD) spectroscopy is a widely‐used method for characterizing individual protein structures in solutions, membranes, films and macromolecular complexes, as well as for probing macromolecular interactions, conformational changes associated with binding substrates, and in different functionally‐related environments. This paper describes a series of related computational and display tools that have been developed over many years to aid in those characterizations and functional interpretations. The new DichroPipeline described herein links a series of format‐compatible data processing, analysis, and display tools to enable users to facilely produce the spectra, which can then be made available in the Protein Circular Dichroism Data Bank (https://pcddb.cryst.bbk.ac.uk/) resource, in which the CD spectral and associated metadata for each entry are linked to other structural and functional data bases including the Protein Data Bank (PDB), and the UniProt sequence data base, amongst others. These tools and resources thus provide the basis for a wide range of traceable structural characterizations of soluble, membrane and intrinsically‐disordered proteins.

## 1 INTRODUCTION

Circular Dichroism (CD) spectroscopy is a structural biology method that has been widely used for protein characterization over many decades (Whitmore and Wallace, 2008). In recent years CD has become a standard biophysical analytical method, especially following the development of quality control standards and new methodologies that have enabled the examination of environmental and substrate‐binding effects on proteins in solution (Wallace, 2020; Miles et al., 2021), in membranes (Miles and Wallace, 2016), and in films (Yoneda et al., 2017), and for identifying structural changes associated with binding of substrates and effector molecules (Wallace and Janes, 2009). New structural analysis algorithms and consistent formatting styles (which enable facile comparisons between data collected under a variety of conditions and in different environments) (Ramalli et al., 2022), novel analytical methods that enable cross‐comparisons with a wide range of biophysical methods (Groves et al., 2021; Miles and Wallace, 2020), and new methods for sequence‐based structure predictions (i.e., Jumper et al., 2021), have enhanced the interoperability and complementarity of CD spectroscopy with other protein characterization methods.

In recent years the availability of high intensity synchrotron radiation light sources has led to the creation of synchrotron radiation circular dichroism (SRCD) beamlines. These have enabled examination of a wider range of sample types, using smaller amounts of precious materials, and exploration of a broader spectral range (including not only UV data but also extending into VUV wavelengths, which have increased the information content of the spectral data obtained) (Wallace and Janes, 2009; Gekko, 2019). As CD and SRCD measurements can be obtained in solution over a range of temperatures and protein concentrations, and under dynamic conditions, both with and without substrate or modulator molecules present, they are now providing valuable complementary information (albeit at lower resolution) to other structure techniques such as X‐ray crystallography, cryo‐electron microscopy, NMR spectroscopy, and solution scattering.

The CD instrumentation developments and enhancements have been accompanied by the creation of a wide range of new computational/bioinformatics methods that can maximize the utility and information content of CD and SRCD spectroscopic measurements. These include bespoke tools for data processing, structural analyses, visual comparisons, cross‐referencing to structural and functional data bases for archiving, and most importantly, public access to CD spectroscopic and associated metadata via the Protein Circular Dichroism Data Bank (PCDDB) (https://pcddb.cryst.bbk.ac.uk) (Ramalli et al., 2022; Whitmore et al., 2017; Whitmore et al., 2011).

**FIGURE 1:**
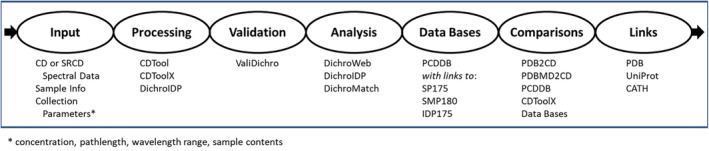
The DichroPipeline. The sequence of functions (top, in circles) and (listed below each of them) the components of the DichroPipeline software involved in these functions.

This paper describes the creation and development of the new “DichroPipeline” (Figure 1), which consists of a series of (format‐compatible) linked versions of existing and new computational resources for CD spectroscopy. They provide an interactive resource for CD data processing, validation, spectral displays, protein structure analyses, a public means for data archiving and retrieval, plus multiple visual and computational methods for comparisons of results from different types of samples and different methodologies.

## 2 TOOLS AND RESOURCES IN THE DICHROPIPELINE

CD spectroscopy is used to characterize soluble, membrane, and intrinsically disordered proteins, including structures that have also proved to be amenable to higher resolution structural studies such as crystallography, NMR spectroscopy, and cryoEM, as well as structures that have not been characterized by these methods. CD has the advantage of being able to explore samples in a wide range of environments, including solutions, membranes, and in dehydrated states (films). The breadth of conditions under which CD data can be collected means it provides an opportunity for comparing structures characterized by different experimental and computational methodologies (Ramalli et al., 2022). This information can now be facilely exploited with the availability of the suite of format‐compatible programs and resources which comprise the DichroPipeline (Figure 1).

DichroPipeline was created to produce simple, comprehensive, and rationalized versions of the CD output data from all sources, and includes as its core element the PCDDB located at https://pcddb.cryst.bbk.ac.uk. The PCDDB contains validated CD and SRCD spectra and associated metadata (Ramalli et al., 2022), and includes cross references/links for each of the entries to sequence (Uniprot) (https://www.uniprot.org/), structure (PDB) (https://www.rcsb.org) and functional databanks, including the Enzyme Commission [E.C.] numbers (https://enzyme.expasy.org/), and CATH classifications (https://www.cathdb.info/). The PCDDB is freely accessible (without requirement for accounts or passwords) for examining and downloading individual protein entries, as well as for complete downloads of its entire contents. However, depositions require password and author verification for traceability reasons. The PCDDB has proven to be a valuable resource for bioinformatics comparisons of individual proteins and groups of proteins, and for the development of new spectroscopic and structural analysis methods. More than 2 million file downloads from the PCDDB have been performed since its creation.

This work describes the contents and functionalities of the elements of the new DichroPipeline, which was created in order to make a simple, comprehensive and rationalized pathway for processing and analyses methods associated with CD and SRCD techniques, and to enhance the utility and ease of use of associated tools and resources for analyses, displays, and traceability of spectral and metadata. All elements of the DichroPipeline use common data formats and units and produce outputs suitable for re‐use with the other components of the Pipeline, and with external tools created by others.

## 3 DATA FORMATS

CD data is most commonly collected using lab‐based “benchtop” instruments (which include many types of commercial instruments with different software and operating systems, as well as a number of bespoke CD instruments or modified instruments created by individual labs). They all tend to include their own data formats (especially the instruments produced by different companies); consequently, an important function of the DichroPipeline is to provide flexibility for input format from different sources, and ultimately to provide a means of converting from bespoke formats to generic output files, which can be used with other analysis methodologies and in comparisons.

On the other hand, many of the SRCD beamlines have adopted a common or generic data output format (unless they incorporate elements of commercially‐produced spectrophotometers, in which case their outputs tend to retain the company‐provided format and units). To rationalize all these different types of data input formats (and units used), the first element in the DichroPipeline targets the concept of compatibility, enabling data collected by different instruments and under different conditions to be facilely displayed, analyzed and compared using a consistent set of software methods, and then output in one of several different commonly‐used formats.

## 4 DATA PROCESSING: CDToolX, CDTool, AND DichroIDP METHODS

CDToolX (Miles and Wallace, 2018) and its predecessor CDTool (Lees et al., 2004) (both available from https://cdtools.cryst.bbk.ac.uk/), have been designed to enable data processing and conversion to CD units and a common ascii output format that is suitable for plotting and use in a range of generic and bespoke analyses for cross‐comparisons and analyses by other user‐provided software and websites. Additionally, the spectral data and associated metadata produced by these methods are output in formats suitable for direct deposition into the Protein Circular Dichroism Data Bank. CDtoolX functions on Windows 10 operating systems while CDTool operates on the older Windows 7 and XP operating systems. Both function on Mac operating systems using a Windows emulator. These tools enable data from different experiments/instruments/conditions to be visually examined for the detection of any possible data anomalies to be investigated prior to undertaking further processing. They provide functions for standard processing methods (addition, averaging, subtraction of baselines, scaling and smoothing), conversion to standard CD units (i.e., mean residue ellipticity (MRE) units based on input protein concentration, and data collection parameters such as the pathlength of data collection cell), and estimation of low wavelength cutoff value for spectral validity. In addition, they can be used for calibration methods based on common standard compounds such as camphorsulfonic acid (CSA) or ammonium camphor sulfonate (ACS). Spectra may also be scaled to each other for graphical comparisons, and, more importantly, the outputs can be used for secondary structure calculations. They can also provide metrics for quality control between different preparations and batches of proteins. Finally, they enable component deconvolution analyses vs. temperature plots, which can be used to highlight possible functionally‐dynamic features/regions of a structure.

DichroIDP (Miles et al., 2023) is a new app created especially for analyses of intrinsically‐disordered proteins (Davey et al., 2019). It is of particular interest as this is a class of proteins that, while functionally very important, generally does not crystallize, nor is it suitable for most types of NMR analyses, so obtaining structural information for these proteins has been challenging until now. However recently, combining theoretical sequence analyses via AlphaFold2 (Jumper et al., 2021) with CD spectral characteristics has enabled the development of algorithms for identifying IDP structural information from their spectra, something not yet possible using other methodologies.

## 5 THE PROTEIN CIRCULAR DICHROISM DATA BANK

The PCDDB (https://pcddb.cryst.bbk.ac.uk/) (Wallace et al., 2003; Whitmore et al., 2017) is a deposition and accession database in which producers of CD (or SRCD) spectra and associated metadata can make their validated results openly accessible to others. It currently (as of August 2023) includes ~750 protein entries. Entries to the database can be made by authors upon registration of their details (for traceability reasons). Users of the data do not need to register for download access, although they have the option to register if they want notification of new entries or features.

## 6 SECONDARY STRUCTURE DATA ANALYSES: DichroWeb

DichroWeb (http://dichroweb.cryst.bbk.ac.uk/html/home.shtml) (Lobley et al., 2002; Whitmore and Wallace, 2004; Miles et al., 2022) is a widely‐used method for calculating secondary structure compositions based on CD data (>1.5 million deconvolutions have been undertaken thus far by 9500+ registered users, the results of which have been cited >7000 times in the literature). It incorporates a range of standard secondary structure analysis methods, with options for using 12 different reference datasets (including bespoke ones for soluble (Lees et al., 2006) and membrane proteins (Abdul‐Gader et al., 2011) and, most recently, for intrinsically‐disordered proteins (IDPs) (Miles et al., 2023).It produces analyses and plots which are directly suitable for inclusions in publications, or for use as a basis for new methodological developments. An archived app version of DichroWeb (DichroWebGit) has recently been made available on GitHub in the PCDDB directory (Table 1) and there are links to Google Colab for running some of these tools. The features and usage of this software are described in detail in Ramalli et al. (2022).

**TABLE 1:** DichroPipeline elements (available either in the individual websites listed at the sites below or in the GitHub repository *(* https://github.com/pcddb).

Online		Website	In GitHub
DichroWeb	P	http://dichroweb.cryst.bbk.ac.uk/html/home.shtm	Y
DichroIDP	I, D	https://dichroidp.cryst.bbk.ac.uk/ (and the IDP database therein)	Y
PCDDB	P, D, DC	https://pcddb.cryst.bbk.ac.uk/	Y
CDtoolX (W10) or CDtool (W7)	D	https://cdtools.cryst.bbk.ac.uk/	Y
	D		
Validichro	I	https://valispec.cryst.bbk.ac.uk/circularDichroism/ValiDichro/upload.html	Y
PDB2CD	I	https://pdb2cd.cryst.bbk.ac.uk/	Y
PDBMD2CD	I	http://pdbmd2cd.cryst.bbk.ac.uk	Y

*Note*: W7 or W10 refers to the version of Windows they were developed for.

Abbreviations: D, downloadable version; DC, downloadable contents; I, interactive, use online; P, requires registration/password for calculations (DW) or for batch submission to the PCDDB database.

## 7 STRUCTURAL COMPARISONS: 2Struc and 2StrucCompare

The 2Struc (Klose et al., 2010) (https://2struc.cryst.bbk.ac.uk/index.php) and 2StrucCompare websites (Drew and Janes, 2019) (https://2strucCompare.cryst.bbk.ac.uk) provide means for visually comparing and examining the secondary structure content of proteins based on their atomic coordinates from crystal, NMR or CryoEM structures. Although not specifically part of the suite of CD‐based analyses programs described above, they can be used to complement the CD‐based secondary structure analyses that form parts of the DichroWeb pipeline. Four different secondary structure analysis packages are provided. 2Struc can be used to determine and display the secondary structure content of a protein. 2StrucCompare enables comparison between two structurally‐related protein chains with the specific aim of identifying small differences between conformations which might appear only as subtle differences in secondary structure features. Such secondary structure differences could be detected in differences between CD spectra, so this program can be utilized to explore and analyze any structural differences that might be the underlying cause of such variations.

## 8 OTHER TOOLS: PDBMD2CD AND PDB2CD

PDBMD2CD (Drew and Janes, 2020) (https://pdbmd2cd.cryst.bbk.ac.uk/) and its predecessor PDB2CD (Mavridis and Janes, 2017) (https://pdb2cd.cryst.bbk.ac.uk/) are tools to generate CD (and SRCD) spectra from protein atomic coordinates. These programs can be especially useful when there is a need to predict what the spectrum of a protein might look like when no such spectrum is available. Spectra generated in this way can be readily compared with the results from the CD analyses of related proteins as a means of offering insight to structural similarities. In addition, PDBMD2CD can be used to generate potential CD spectra for calculated structures produced by molecular dynamics (MD) simulations. Comparisons of these generated spectra with experimentally‐obtained spectra can offer an opportunity to further explore the suitability of existing methods and for the developing of new MD methods.

## 9 DichroIDP: ANALYSES OF INTRINSICALLY‐DISORDERED PROTEINS

DichroIDP (https://dichroidp.cryst.bbk.ac.uk/) is a new (Miles et al., 2023) bespoke methodology for analyzing IDPs or proteins with Intrinsically Disordered Regions (IDRs), which can play important roles in regulatory functions. These proteins are not primarily composed of the canonical helix, sheet and turn structures typically found in ordered proteins, but have significant numbers of residues (often the functionally‐important ones) which have “disordered” structures not normally identified in calculations of secondary structures. For the most part, these types of proteins do not crystallize, so spectroscopic characterizations can be essential. The DichroIDP methodology can be used to identify such disordered structures on the basis of their CD spectra. Because there are many types of disorder (static, dynamic, flexible), DichroIDP utilizes the SELCON3 (Sreerema and Woody, 1993) algorithm and several reference databases for analyses of the sequences and spectra of ordered and disordered regions within IDPs.

## 10 ValiDichro TOOLS: DATA VALIDATION

The ValidDichro website (https://valispec.cryst.bbk.ac.uk/circularDichroism/ValiDichro/upload.html) (Woollett et al., 2013) includes procedures for data quality control and parameter checking of entries to be deposited in the PCDDB. It includes 25 tests for data completeness, consistency, and quality. The report produced for the depositor includes suggestions for remedying any possible issues identified before the file is submitted to the PCDDB. Primarily developed as a quality‐control aid for depositions to the PCDDB, it can also be used (without requirement for user registration) as a guide to experimentalists for checking their data quality prior to using in analyses or publications. Passing the standards in ValiDichro is a required quality control element for depositions to the PCDDB. These reports are available for users of the PCDDB to examine and download.

## 11 CONCLUSIONS

Over the past three decades, we have created a wide range of tools and resources for CD and SRCD spectroscopy. This paper describes the rationalization of the computational and methodological elements in the new DichroPipeline resource, enabling their widespread use, quality control, and documentation for the characterization of proteins. In addition, the linked information available through the DichroPipeline enables facile comparisons of circular dichroism spectroscopic characterizations of proteins with other structural and functional information.

## AUTHOR CONTRIBUTIONS

Both authors contributed equally to this article. They have worked together for more than 30 years, including supervising the students and postdocs whose names appear with them as authors in the cited works.

## Data Availability

The above tools are free and accessible to all (academic) users. Some may require user registration.
